# Impaired Glucose Metabolism in Young Patients with First-episode Schizophrenia Aged from 16 to 35 Years

**DOI:** 10.2174/011570159X358856250220114527

**Published:** 2025-10-22

**Authors:** Jing Yao, Nan Chen, Meihong Xiu, Fengchun Wu, Yuanyuan Huang

**Affiliations:** 1 Peking University HuiLongGuan Clinical Medical School, Beijing Huilongguan Hospital, Beijing, China;; 2 Department of Psychiatry, The Affiliated Brain Hospital, Guangzhou Medical University, Guangzhou, China;; 3 Guangdong Engineering Technology Research Center for Translational Medicine of Mental Disorders, Guangzhou, China;; 4 Key Laboratory of Neurogenetics and Channelopathies of Guangdong Province and the Ministry of Education of China, Guangzhou Medical University, Guangzhou, China

**Keywords:** Schizophrenia, glucose metabolism, sex difference, HOMA-IR, early stage of psychosis, insulin resistance

## Abstract

**Background:**

Previous studies in drug-naïve, first-episode patients with schizophrenia (FES) suggest glucose metabolism differences in the early stage of psychosis. However, we have limited knowledge of abnormalities in glucose metabolism in young and drug-naïve FES patients.

**Methods:**

A total of 162 drug-naïve patients with schizophrenia (aged from 18 to 35 years) and 110 age-, sex-matched healthy control subjects were enrolled. Fasting glucose, fasting insulin, glycated hemoglobin (HbA1c), and insulin resistance (HOMA-IR) index were measured in patients and control subjects.

**Results:**

We found that young drug-naïve FES patients exhibited abnormal glucose metabolism compared with control subjects. Fasting insulin, fasting glucose, and HOMA-IR were higher in patients than in controls (all *p<*0.05). In addition, female patients had significantly higher fasting insulin levels and HOMA-IR than male patients (both *p<*0.05), as well as in the healthy controls. Binary logistic regression analysis further identified that smoking status, HOMA-IR, and HbA1c were the contributing factors to schizophrenia, after controlling for age and sex.

**Conclusion:**

This study suggests abnormal glucose metabolism in young drug-naïve FES patients, highlighting that these glucose metabolic issues are present at the very early stage of the disease. The identification of abnormal glucose metabolism at the early stages of schizophrenia provides insights into the biological underpinnings of schizophrenia and may lead to more targeted interventions for patients in the early stages of the disease.

## 1 INTRODUCTION

Schizophrenia (SZ) is a serious mental illness. Studies have reported that individuals with severe mental illness have almost twice the risk of dying from cardiovascular disease than the risk in the general population. Patients with SZ die 12-15 years earlier than the general population [1-4]. Patients are at risk of premature mortality due to causes unrelated to the central nervous system but correlated with cardiometabolic risk factors such as metabolic syndrome and type 2 diabetes [5-7]. Evidence showed that the high prevalence of cardio-metabolic risk factors in SZ is associated with adverse effects of antipsychotic medication or the disease itself. The link between SZ and diabetes was identified as early as the 19^th^ century or the beginning of the 20^th^ century, long before the advent of antipsychotic medications, during an era when diets did not typically cause metabolic disorders [8]. This historical context indicates that the link between schizophrenia and metabolic disorders is not solely due to modern pharmaceutical treatments but has deeper roots.

Indeed, recent studies have reported impaired glucose metabolism, and type 2 diabetes in first-episode patients with affective and non-affective psychosis without or with minimal antipsychotic exposure [9-11], suggesting that there were changes in glucose metabolism homeostasis at the onset of psychosis. For example, Perry *et al*. showed a potential relationship between first-episode psychosis and impaired glucose tolerance and insulin resistance in a system review and meta-analysis [10]. On the other hand, Pillinger *et al*. found in another meta-analysis that, compared with controls, drug-naïve and BMI-matched patients, diet and exercise-matched patients, or race/ethnicity-matched patients with schizophrenia had increased fasting glucose levels, fasting glucose after an oral glucose tolerance test, and insulin resistance [12]. A recent meta-analysis of antipsychotic-naïve patients with first-episode psychosis reported an increased incidence of metabolic syndrome in drug-naïve first-episode patients [13]. These findings support that SZ itself, independent of medication, lifestyle, or racial/ethnic factors, is associated with metabolic abnormalities. Notably, a meta-analysis from Bartolomeis *et al*. in preclinical studies revealed that the brain neurotransmitter system is susceptible to disturbed insulin signaling, possibly contributing to the association between schizophrenia and glucose metabolism abnormalities [14]. This implies a potential biological mechanism linking metabolic abnormalities with the pathophysiology of SZ.

Although the patients in the above studies were all first-episode, it is noteworthy that the age range of the participants was quite wide, extending from 16 to 50 years old. It is well-known that younger patients had lower rates of glucose metabolism abnormalities compared to older patients, highlighting the importance of considering age-related metabolic differences when evaluating and treating SZ. Currently, the glucose metabolism abnormalities in patients with SZ aged from 18 to 35 years old have not been focused on. Further research is warranted to explore the association between age, glucose metabolism, and SZ, with a particular focus on younger subgroups, to develop more tailored and effective treatment strategies. In the present study, young drug-naïve first-episode SZ (FES) patients (aged from 18 to 35 years) were recruited to examine: 1) the rate of abnormal glucose metabolism in young drug-naive FES patients; and 2) gender differences in the prevalence of glucose metabolism abnormality in patients.

## 2 METHODS

### 2.1 Samples

A total of 162 young patients (male/female=86/76) diagnosed with SZ by experienced psychiatrists using the *DSM-V* were recruited in four psychiatric hospitals. The inclusion criteria included: 1) 18-35 years; 2) disease duration < 60 months; 3) first episode; 4) drug-naïve or accumulative for less than 2 weeks; and 5) male or female. Exclusion criteria were: (1) organic brain disorder; (2) abuse or drug dependence; (3) pregnant or breastfeeding women; and 4) no major medical comorbidities, including cancer, cerebrovascular disease, or hypertension.

A total of 110 healthy subjects (male/female=62/48) (aged from 18 to 35 years) were recruited from the community through advertisement. An experienced psychiatrist interviewed all participants according to the SCID to screen for a current or past history of diagnosis of major Axis I disorders. They were also screened for a family history of mental disease through interviews.

This study was approved by the Ethics Committee of the hospital (Ethic No. 2011-4). Each patient signed the written informed consent.

### 2.2 Measurement of Biomarkers

Fasting blood samples were collected from each patient between 7:00 AM and 8:00 AM. The fasting glucose (FG) level was determined in the laboratory using an enzymatic method. The reference range for FG levels in the general population was 3.9-6.1 mmol/L. The fasting insulin (FI) level and HbA1c level were determined using the kits. The normal reference range for FI and HbA1c was 2.6-24.9 μU/mL and 4.8-5.9%, respectively. The insulin resistance (HOMA-IR) index was calculated using a formula in a previous study [15]. The normal reference range of HOMA-IR did not exceed 2.69. Glucose metabolism parameters were analyzed in duplicate by three experienced technicians.

Body mass index (BMI) was calculated as weight over squared height (m^2^). Height was recorded to the nearest centimeter. Weights were recorded to the nearest half kilogram. The normal reference range for BMI was 18.5-24.0 kg/m^2^.

### 2.3 Statistical Analysis

All continuous variables were expressed as mean ± standard deviation (SD) and categorical variables as n (%). ANOVA and *X*^2^ tests were performed to evaluate the data between patients and controls or between men and women. Pearson’s correlation analysis was performed to analyze the association between demographic and clinical characteristics and glucose metabolism parameters in patients and controls, or male and female subgroups. Lastly, the logistic regression analysis was used to test for differences in the contribution of glucose metabolism parameters as well as demographic and clinical characteristics to SZ. The area under the receiver operating characteristics (AUCROC) was performed to determine the discriminatory capacity of significant glucose metabolic parameters to distinguish between patients and controls.

Bonferroni corrections were performed for multiple comparisons to reduce the risk of a Type I error in this study. We applied the Bonferroni correction to adjust the significance level when comparing 5 glucose metabolic parameters between groups. The original significance level was divided by the number of comparisons and yielded an α of .05/5 = 0.01 as a new threshold for significance.

## 3 RESULTS

### 3.1 Comparison of Glucose Metabolism Parameters between Patients and Controls

The mean age and disease duration of all young FES patients were 24.2 ± 6.1 years and 28.5 ± 20.4 months, respectively. There were no significant differences between patients and control subjects in terms of age, sex, smoking status, and years of education (all *p >* 0.05) (Table **1**). Significant differences were observed in BMI, FG, FI, HAbc1, and HOMR1 between the patient and control groups (*p =* 0.009,

### 3.2 Sex Differences in Glucose Metabolism Parameters

We found no significant differences in age and disease duration between men and women in the patient group (all *p >* 0.05). Table **1** shows the demographic, clinical data, and levels of glucose metabolism parameters in total patients or males and females respectively. The mean values of glucose metabolism parameters among patients were within the normal reference range (Table **1**). Men had higher BMI values than women (F=4.7, *p =* 0.03). Female patients had higher FI levels (F=5.1, *p =* 0.03) and HOMA-IR index (F=4.1, *p =* 0.04) than male patients.

According to the reference values for glucose metabolism parameters in the general population, the rates of abnormal glucose metabolism in young patients were analyzed. 72 patients (37 males and 35 females) had abnormal BMI values, among which 20 were lower than the reference values and 52 were above the normal values. We found no significant differences in the rates of abnormal BMI between male and female patients (43% *vs.* 43.1%, *X*^2^=0.15, *p =* 0.75). FI levels were not in the normal reference value in 7 patients (2 male and 5 female). FG levels were higher than normal reference values in 2 male patients. One male patient had higher HbA1c values than the reference values. HOMA-IR was higher than reference values in 73 patients (37 males and 36 females). No significant difference was observed in the rates of abnormal FI, FG, HOMA-IR, and HbA1c between males and females (all *p >* 0.05).

In control subjects, there were no significant differences in the five glucose metabolism parameters between males and females (all *p >* 0.05).

### 3.3 Relationships of Glucose Metabolism Parameters with SZ

In all patients, Pearson correlation analysis revealed significant correlations of age with BMI (r= 0.30, *p <* 0.001) and FG (r= 0.32, *p <* 0.001). In addition, onset age was associated with BMI (r= 0.29, *p <* 0.001) and FG (r= 0.30, *p <* 0.001). We then analyzed the correlations separately for males and females and found that the correlations existed in both male patients and female patients (Table **2**). In control subjects, age was correlated with BMI only in males (r= 0.29, *p =* 0.02).

Logistic regression analysis was used to analyze the association between glucose metabolic parameters and SZ, with demographics and glucose metabolism as dependent variables and diagnosis (patient vs control) as independent variables. We found that HbA1c (β=0.88, *X*^2^=5.27, OR=2.40, 95% CI: 1.14-5.07, *p =* 0.02), HOMA-IR (β=-4.57, *X*^2^=5.00, OR=0.01, 95% CI: 0.0002-0.57 *p =* 0.025), and smoking status (β=-0.99, *X*^2^=4.72, OR=0.37, 95% CI: 0.15-0.91, *p =* 0.03) were the independent contributors to SZ (Table **3**). Furthermore, AUCROC showed the following values for each risk factor: HOMA-IR was 0.691 (95% CI: 0.629, 0.752, *p <* 0.001), HbA1c was 0.457 (95% CI: 0.385, 0.529, *p =* 0.229), and smoking status was 0.530 (95% CI: 0.461, 0.600, *p =* 0.397). Finally, we found that HOMA-IR had a higher AUC value of 0.691 to distinguish patients and controls.

## 4 DISCUSSION

In this study, we found that: 1) abnormal glucose metabolism parameters in young adult patients with drug-naïve FES compared with healthy control subjects; 2) higher FI levels and HOMA-IR index in females compared to males; and 3) after controlling for confounding factors, smoking status, HOMA-IR and HbA1c were key factors contributing to the development of SZ.

We found that glucose metabolism parameters were abnormal in young FES patients with SZ compared with healthy control subjects, which is in line with previous studies [9, 10, 13, 16-21]. In particular, recent meta-analyses also demonstrated higher insulin resistance, HbA1c, and FI after oral glucose tolerance test (OGTT) in drug-naïve FES patients compared with control subjects [9, 10, 12], highlighting abnormal glucose metabolism in SZ patients at the onset of the first psychotic episode. In addition, studies in adolescent or young FES patients (8-23 years) have also found higher levels of FG, HbA1c, and HOMA-IR than healthy controls [22, 23]. However, two meta-analyses reported no significant difference in FG levels between patients and controls [10, 12], which was inconsistent with our findings. On the other hand, although there was a difference in the HbA1c levels of the patients compared to the controls, this study found that the difference was no longer significant after Bonferroni correction, suggesting that the difference between the patients and the controls appears small. We speculate that the discrepancy between studies may be related to age-related physiological and biological changes (*e.g*., levels of oxidative stress, inflammation, and cytotoxicity) or patient characteristics (*e.g*., sex dimorphism, relationship with family members, education, diet, exercise, and social activities). Notably, our study focused on young drug-naïve patients with SZ (16-35 years), whereas other studies recruited adolescents aged 12-17 years or young patients aged 8-23 years, or patients with a wide range of ages, which was the possible reason for the differences between the studies. Our findings provide a deeper understanding of the metabolic abnormality in individuals with FES, which is important for both diagnosis and treatment strategies.

Particularly, previous and our studies demonstrated that FI and HOMA-IR were consistently reported to be altered in young patients with SZ [12, 15, 24], indicating that increases in FI and HOMA-IR are common changes in the early stage of SZ and are more sensitive to detecting metabolic abnormalities in SZ compared to other measures. Currently, the relationship between insulin signaling and SZ is complex and remains unclear. Dysregulation of insulin signaling has been associated with the pathophysiology of SZ in FES patients [14, 25]. Insulin receptors are widely expressed in the hippocampus, a region that is related to learning, memory, and other cognition [26]. Insulin signaling can regulate brain metabolism and energy homeostasis [27-29]. It can regulate synaptic plasticity, and circuit connectivity through the PI3K/Akt/mTOR pathway, which is a key cellular signaling pathway involved in cell growth [28, 29]. Insulin resistance is also associated with increased inflammation [30, 31]. It has been shown that chronic inflammation is one of the major causes of insulin resistance and that inflammatory mediators can induce insulin resistance by affecting insulin signaling in adipose tissue [32, 33]. Specifically, the signaling pathway of the transcription factor NF-κβ plays a key role in insulin sensitivity and M1/M2-type macrophage polarization also plays an important role in insulin resistance [34]. Moreover, anti-inflammatory and neuroprotective roles for insulin signaling in the CNS have also been demonstrated [35-37]. Inflammation may be a common mechanism between insulin resistance and SZ and may play a bridging role between the two pathological states [32].

Interestingly, recent evidence reported that an impact of early life events, for example, birth weight (BW), an indirect marker of the fetal environment, has been associated with impaired glucose metabolism in the general population over time [38]. Prior exposure to prenatal stress may trigger epigenetic changes involved in the fetal metabolic programming of SZ. The interaction between the hereditary and environmental factors together precipitates disease onset by disrupting the trajectory of normal brain development, *via* a “thrifty” phenotype [39]. Therefore, we speculate that in the early stage of SZ, a critical stage of neurodevelopment, impaired insulin signaling may have a deleterious effect on the neuronal functions of SZ.

Another finding of this study was that there were sex differences in glucose metabolic parameters in young FES patients with SZ and that FI and HOMR-IR were higher in females than males. Our findings were consistent with a recent meta-analysis in first-episode psychosis, which reported first-episode patients had more severe metabolic changes compared with control subjects, and female sex was associated with worse metabolic outcomes [40]. This meta-analysis provides a broader measurement of abnormal metabolic parameters, including glucose post-OGTT, total cholesterol, low-density lipoprotein (LDL)-cholesterol, and high-density lipoprotein (HDL)-cholesterol. Insulin resistance has been reported to be associated with obesity and high FI [41]. Even though BMI values were higher in males than females in the current study, we found no sex difference in the rate of abnormal BMI in patients. Thus, BMI, an important aspect of metabolic health, did not appear to be the cause of a significant influence of sex on glucose metabolism in this study. Sex differences in metabolism are well-documented and can be attributed to various biological factors, including hormonal differences, body composition, and genetic factors [42-44]. Hormones such as estrogen and testosterone can influence insulin sensitivity and glucose metabolism through a variety of mechanisms, including direct effects on mitochondrial function, regulation of dopamine activity, and interaction with insulin receptors [45, 46]. For example, estrogens (especially estradiol E2) protect pancreatic β-cells from apoptosis, stimulate insulin secretion, and prevent diet-induced insulin resistance [47]. Estrogen increases the body's sensitivity to insulin and decreases insulin resistance in peripheral tissues. On the other hand, estrogen receptor stimulation inhibits adipogenesis in β-cells and limits the negative effects of excess fat in the cells. In summary, estrogen positively affects pancreatic β-cell function and insulin resistance through these mechanisms. Similarly, testosterone also plays an important role in the regulation of insulin sensitivity and glucose metabolism. Testosterone physiologically enhances glucose-stimulated insulin secretion, increases glucagon-like peptide-1 receptor (GLP-1) action, and reduces inflammation, thereby maintaining β-cell functions [48]. Severe testosterone deficiency has been reported to be associated with the development of insulin resistance. Indeed, estrogen and testosterone have been described to be reduced in drug-naïve patients with psychosis [49, 50].

However, we also found sex differences in FI and HOMR-IR in young healthy control subjects, suggesting that the difference in FI and HOMR-IR values may be partially due to inherent biological and physiological differences between males and females. Factors influencing insulin resistance and glucose metabolism may be also related to sex hormones, genetic factors, and general health status rather than only to the presence of SZ.

Several limitations should be noted in this study. First, this study does not account for important lifestyle factors such as diet, physical activity, or other health behaviors that could significantly affect glucose metabolism. This is a major limitation, which should be considered in further research to elucidate the link between SZ and insulin signaling. Second, this is a cross-sectional study and data was collected at only one time point, which was useful for obtaining a quick understanding of the prevalence of glucose metabolism abnormalities at the very onset of psychosis. However, this design limits the ability to infer causality and provides limited insight into the progression of diseases and how glucose metabolism changes as the disease progresses. It would be interesting to examine longitudinal changes in glucose metabolism concerning SZ, according to a longitudinal study following patients over time.

## CONCLUSION

This study revealed abnormal glucose metabolism in young adult drug-naïve FES patients with SZ compared with healthy controls, suggesting that there may be a link between SZ and glucose metabolism. Female patients had higher FI levels and HOMA-IR index than male patients, suggesting that female patients had a higher degree of insulin resistance, which is a risk factor for developing type 2 diabetes. These findings suggest that young, drug-naïve FES patients should be monitored for glucose metabolic disorders, especially if they are female. Additionally, further research into understanding the relationship between SZ and glucose metabolism may lead to better treatment strategies to address both the mental health and metabolic health of these patients.

## Figures and Tables

**Table 1:** The demographic and glucose metabolism in young patients with schizophrenia.

**-**	**Total Sample** **n = 162**	**Healthy Controls** **n = 110**	**Male Patients** **n = 86**	**Female Patients** **n = 76**
Age (ys)	24.2 ± 6.1	24.4 ± 6.3	23.5 ± 5.3	24.9 ± 6.8
Education (ys)	11.9 ± 3.5	11.8 ± 3.0	12.0 ± 3.5	11.8 ± 3.7
**Smoke Status**				
Yes	26(16.0%)	11(10.0%)	23(26.7%)	3(3.9%)
No	136(84.0%)	99(90.0%)	63(73.3%)	73(96.1%)
Disease duration (ms)	26.8 ± 20.2	-	28.2 ± 21.2	25.3 ± 18.9
Onset age (ys)	22.0 ± 6.0	-	21.1 ± 5.0	23.0 ± 6.8
**Glucose Metabolism Parameters**				
BMI (kg/m^2^)	22.3 ± 3.4	21.4 ± 2.3*	22.9 ± 3.4	21.7 ± 3.2
FG	5.2 ± 0.4	5.0 ± 0.5*	5.2 ± 0.4	5.1 ± 0.4
FI	11.7 ± 6.1	8.2 ± 3.1*	10.7 ± 6.0	12.8 ± 6.0
HAbc1	5.3 ± 0.3	5.4 ± 0.6*	5.3 ± 0.3	5.2 ± 0.3
HOMRI	2.7 ± 1.5	1.8 ± 0.7*	2.5 ± 1.4	3.0 ± 1.5

**Abbreviations**: ys years, ms months, BMI body mass index, FG fasting glucose, FI fasting insulin, HOMRI insulin resistance index.

**Note**: *p < 0.05. *p =* 0.007, *p =* 0.03, *p* < 0.001, *p* < 0.001). BMI, FG, FI, and HOMR1 were higher in patients compared to healthy controls. After Bonferroni correction, the differences in BMI, FG, FI, and HOMR1 remained significant (*p* <p_Bonferroni_= 0.01).

**Table 2:** Associations between demographic and clinical variables and glucose metabolism parameters in male and female FES patients.

**-**	**-**	**BMI**	**FG**	**HbA1c**	**FI**	**HOMA-IR**
**Male FES Patients (n = 86)**						
Age	r	0.41	0.41	0.05	0.02	0.07
	p	<0.001	<0.001	0.65	0.82	0.50
Onset age	r	0.23	0.40	-0.004	0.03	0.08
	p	0.03	<0.001	0.97	0.76	0.47
Disease duration	r	0.02	0.08	0.14	-0.04	-0.03
	p	0.82	0.48	0.19	0.71	0.81
**Female FES Patients (n = 76)**						
Age	r	0.12	0.15	0.01	0.10	-0.03
	p	0.29	0.18	0.93	0.41	0.80
Onset age	r	0.05	0.17	-0.02	0.08	-0.04
	p	0.66	0.14	0.89	0.47	0.76
Disease duration	r	0.10	-0.08	0.04	0.01	-0.01
	p	0.69	0.71	0.37	0.71	0.47

**Abbreviations**: BMI body mass index; FG fasting glucose, FI fasting insulin.

**Table 3:** Logistic regression of glucose metabolism parameter levels related to sex difference in patients.

**-**	**β**	**Std.Error**	**Wals**	**Sig.**	**OR (95% CI)**
(Constant) Sex Age (ys) Smoking status	-4.867	4.643	1.099	0.295	0.008
	-0.371	0.317	1.365	0.243	0.690(0.371, 1.286)
	0.042	0.026	2.651	0.103	1.043(0.991, 1.097)
	-0.992	0.457	4.717	0.030	0.371(0.152, 0.908)
**Glucose Metabolism Parameters**					
BMI (kg/m^2^)	-0.086	0.054	2.579	0.108	0.917(0.826, 1.019)
FG	0.541	0.818	0.438	0.508	1.718(0.346, 8.543)
FI	0.883	0.462	3.664	0.056	2.419(0.979, 5.977)
HAbc1	0.875	0.381	5.274	0.022	2.400(1.137, 5.065)
HOMRI	-4.569	2.043	5.000	0.025	0.010(0.0002, 0.569)

**Abbreviation**: ys years.

## Data Availability

The data and supportive information are available within the article.

## LIST OF ABBREVIATIONS

BW: Birth Weight
FG: Fasting Glucose
FI: Fasting Insulin
HDL: High-density Lipoprotein
LDL: Low-density Lipoprotein
OGTT: Oral Glucose Tolerance Test
SD: Standard Deviation
SZ: Schizophrenia

## References

[1] Fleishhacker W.W., Cetkovich-Bakmas M., De Hert M., Hennekens C.H., Lambert M., Leucht S., Maj M., McIntyre R.S., Naber D., Newcomer J.W., Olfson M., Ösby U., Sartorius N., Lieberman J.A. (2008). Comorbid somatic illnesses in patients with severe mental disorders: Clinical, policy, and research challenges.. J. Clin. Psychiatry.

[2] Lawrence D.M., D’Arcy C., Holman J., Jablensky A.V., Hobbs M.S.T. (2003). Death rate from ischaemic heart disease in Western Australian psychiatric patients 1980-1998.. Br. J. Psychiatry.

[3] Saha S., Chant D., McGrath J. (2007). A systematic review of mortality in schizophrenia: Is the differential mortality gap worsening over time?. Arch. Gen. Psychiatry.

[4] De Hert M., Schreurs V., Vancampfort D., Van Winkel R. (2009). Metabolic syndrome in people with schizophrenia: A review.. World Psychiatry.

[5] Ösby U., Correia N., Brandt L., Ekbom A., Sparén P. (2000). Mortality and causes of death in schizophrenia in Stockholm County, Sweden.. Schizophr. Res..

[6] Crump C., Winkleby M.A., Sundquist K., Sundquist J. (2013). Comorbidities and mortality in persons with schizophrenia: A Swedish national cohort study.. Am. J. Psychiatry.

[7] Laursen T.M., Nordentoft M., Mortensen P.B. (2014). Excess early mortality in schizophrenia.. Annu. Rev. Clin. Psychol..

[8] Kohen D. (2004). Diabetes mellitus and schizophrenia: Historical perspective.. Br. J. Psychiatry.

[9] Greenhalgh A.M., Gonzalez-Blanco L., Garcia-Rizo C., Fernandez-Egea E., Miller B., Arroyo M.B., Kirkpatrick B. (2017). Meta-analysis of glucose tolerance, insulin, and insulin resistance in antipsychotic-naïve patients with nonaffective psychosis.. Schizophr. Res..

[10] Perry B.I., McIntosh G., Weich S., Singh S., Rees K. (2016). The association between first-episode psychosis and abnormal glycaemic control: Systematic review and meta-analysis.. Lancet Psychiatry.

[11] Garcia-Rizo C., Kirkpatrick B., Fernandez-Egea E., Oliveira C., Meseguer A., Grande I., Undurraga J., Vieta E., Bernardo M. (2014). “Is bipolar disorder an endocrine condition?” Glucose abnormalities in bipolar disorder.. Acta Psychiatr. Scand..

[12] Pillinger T., Beck K., Gobjila C., Donocik J.G., Jauhar S., Howes O.D. (2017). Impaired glucose homeostasis in first-episode schizophrenia: A systematic review and meta-analysis.. JAMA Psychiatry.

[13] Garrido-Torres N., Rocha-Gonzalez I., Alameda L., Rodriguez-Gangoso A., Vilches A., Canal-Rivero M., Crespo-Facorro B., Ruiz-Veguilla M. (2021). Metabolic syndrome in antipsychotic-naïve patients with first-episode psychosis: A systematic review and meta-analysis.. Psychol. Med..

[14] de Bartolomeis A., De Simone G., De Prisco M., Barone A., Napoli R., Beguinot F., Billeci M., Fornaro M. (2023). Insulin effects on core neurotransmitter pathways involved in schizophrenia neurobiology: A meta-analysis of preclinical studies. Implications for the treatment.. Mol. Psychiatry.

[15] Xiu M., Fan Y., Liu Q., Chen S., Wu F., Zhang X. (2023). Glucose metabolism, hippocampal subfields and cognition in first-episode and never-treated schizophrenia.. Int. J. Clin. Health Psychol..

[16] Thakore J.H. (2004). Metabolic disturbance in first-episode schizophrenia.. Br. J. Psychiatry.

[17] Mitchell A.J., Vancampfort D., De Herdt A., Yu W., De Hert M. (2013). Is the prevalence of metabolic syndrome and metabolic abnormalities increased in early schizophrenia? A comparative meta-analysis of first episode, untreated and treated patients.. Schizophr. Bull..

[18] Arranz B., Rosel P., Ramírez N., Dueñas R., Fernández P., Sanchez J.M., Navarro M.A., San L. (2004). Insulin resistance and increased leptin concentrations in noncompliant schizophrenia patients but not in antipsychotic-naive first-episode schizophrenia patients.. J. Clin. Psychiatry.

[19] Misiak B., Łaczmański Ł., Słoka N.K., Szmida E., Piotrowski P., Loska O., Ślęzak R., Kiejna A., Frydecka D. (2016). Metabolic dysregulation in first-episode schizophrenia patients with respect to genetic variation in one-carbon metabolism.. Psychiatry Res..

[20] Ryan M.C.M., Collins P., Thakore J.H. (2003). Impaired fasting glucose tolerance in first-episode, drug-naive patients with schizophrenia.. Am. J. Psychiatry.

[21] Spelman L.M., Walsh P.I., Sharifi N., Collins P., Thakore J.H. (2007). Impaired glucose tolerance in first-episode drug-naïve patients with schizophrenia.. Diabet. Med..

[22] Jensen K.G., Correll C.U., Rudå D., Klauber D.G., Stentebjerg-Olesen M., Fagerlund B., Jepsen J.R.M., Fink-Jensen A., Pagsberg A.K. (2017). Pretreatment cardiometabolic status in youth with early-onset psychosis.. J. Clin. Psychiatry.

[23] Petruzzelli M.G., Margari M., Peschechera A., de Giambattista C., De Giacomo A., Matera E., Margari F. (2018). Hyperprolactinemia and insulin resistance in drug naive patients with early onset first episode psychosis.. BMC Psychiatry.

[24] Yang W., Zheng L., Zheng B., Zeng S., Li J., Liang B., Zhu J., Zhang M. (2020). A meta-analysis of abnormal glucose metabolism in first-episode drug-naive schizophrenia.. Psychiatr. Danub..

[25] Chouinard V.A., Henderson D.C., Dalla Man C., Valeri L., Gray B.E., Ryan K.P., Cypess A.M., Cobelli C., Cohen B.M., Öngür D. (2019). Impaired insulin signaling in unaffected siblings and patients with first-episode psychosis.. Mol. Psychiatry.

[26] Deng W., Aimone J.B., Gage F.H. (2010). New neurons and new memories: How does adult hippocampal neurogenesis affect learning and memory?. Nat. Rev. Neurosci..

[27] Zhao W.Q., Alkon D.L. (2001). Role of insulin and insulin receptor in learning and memory.. Mol. Cell. Endocrinol..

[28] Milstein J.L., Ferris H.A. (2021). The brain as an insulin-sensitive metabolic organ.. Mol. Metab..

[29] García-Cáceres C., Quarta C., Varela L., Gao Y., Gruber T., Legutko B., Jastroch M., Johansson P., Ninkovic J., Yi C.X., Le Thuc O., Szigeti-Buck K., Cai W., Meyer C.W., Pfluger P.T., Fernandez A.M., Luquet S., Woods S.C., Torres-Alemán I., Kahn C.R., Götz M., Horvath T.L., Tschöp M.H. (2016). Astrocytic insulin signaling couples brain glucose uptake with nutrient availability.. Cell.

[30] Shoelson S.E., Lee J., Goldfine A.B. (2006). Inflammation and insulin resistance.. J. Clin. Invest..

[31] Wu H., Ballantyne C.M. (2020). Metabolic inflammation and insulin resistance in obesity.. Circ. Res..

[32] Zand H., Morshedzadeh N., Naghashian F. (2017). Signaling pathways linking inflammation to insulin resistance.. Diabetes Metab. Syndr..

[33] Day C.P. (2006). From fat to inflammation.. Gastroenterology.

[34] Suryavanshi S.V., Kulkarni Y.A. (2017). NF-κβ: A potential target in the management of vascular complications of diabetes.. Front. Pharmacol..

[35] Spielman L.J., Klegeris A. (2014). The role of insulin and incretins in neuroinflammation and neurodegeneration.. Immunoendocrinology (Houst.).

[36] Dandona P., Chaudhuri A., Ghanim H., Mohanty P. (2009). Insulin as an anti-inflammatory and antiatherogenic modulator.. J. Am. Coll. Cardiol..

[37] Ferreira S.T., Clarke J.R., Bomfim T.R., De Felice F.G. (2014). Inflammation, defective insulin signaling, and neuronal dysfunction in Alzheimer’s disease.. Alzheimers Dement..

[38] Garcia-Rizo C., Cabrera B., Bioque M., Mezquida G., Lobo A., Gonzalez-Pinto A., Diaz-Caneja C.M., Corripio I., Vieta E., Baeza I., Garcia-Portilla M.P., Gutierrez-Fraile M., Rodriguez-Jimenez R., Garriga M., Fernandez-Egea E., Bernardo M. (2022). The effect of early life events on glucose levels in first-episode psychosis.. Front. Endocrinol. (Lausanne).

[39] Garcia-Rizo C., Bitanihirwe B.K.Y. (2020). Implications of early life stress on fetal metabolic programming of schizophrenia: A focus on epiphenomena underlying morbidity and early mortality.. Prog. Neuropsychopharmacol. Biol. Psychiatry.

[40] Pillinger T., McCutcheon R.A., Howes O.D. (2023). Variability of glucose, insulin, and lipid disturbances in first-episode psychosis: A meta-analysis.. Psychol. Med..

[41] Friedrich N., Thuesen B., Jørgensen T., Juul A., Spielhagen C., Wallaschofksi H., Linneberg A. (2012). The association between IGF-I and insulin resistance: A general population study in Danish adults.. Diabetes Care.

[42] Abel K.M., Drake R., Goldstein J.M. (2010). Sex differences in schizophrenia.. Int. Rev. Psychiatry.

[43] da Silva T.L., Ravindran A.V. (2015). Contribution of sex hormones to gender differences in schizophrenia: A review.. Asian J. Psychiatr..

[44] Riecher-Rössler A., Häfner H. (2000). Gender aspects in schizophrenia: Bridging the border between social and biological psychiatry.. Acta Psychiatr. Scand..

[45] Mauvais-Jarvis F., Clegg D.J., Hevener A.L. (2013). The role of estrogens in control of energy balance and glucose homeostasis.. Endocr. Rev..

[46] Kelly D.M., Jones T.H. (2013). Testosterone: A metabolic hormone in health and disease.. J. Endocrinol..

[47] Alemany M. (2021). Estrogens and the regulation of glucose metabolism.. World J. Diabetes.

[48] Kautzky-Willer A., Leutner M., Harreiter J. (2023). Sex differences in type 2 diabetes.. Diabetologia.

[49] Fernandez-Egea E., García-Rizo C., Miller B., Parellada E., Justicia A., Bernardo M., Kirkpatrick B. (2011). Testosterone in newly diagnosed, antipsychotic-naive men with nonaffective psychosis: A test of the accelerated aging hypothesis.. Psychosom. Med..

[50] Brand B.A., de Boer J.N., Sommer I.E.C. (2021). Estrogens in schizophrenia: Progress, current challenges and opportunities.. Curr. Opin. Psychiatry.

